# WHO consultation on group B *Streptococcus* vaccine development: Report from a meeting held on 27–28 April 2016

**DOI:** 10.1016/j.vaccine.2016.12.029

**Published:** 2019-11-28

**Authors:** Miwako Kobayashi, Stephanie J. Schrag, Mark R. Alderson, Shabir A. Madhi, Carol J. Baker, Ajoke Sobanjo-ter Meulen, David C. Kaslow, Peter G. Smith, Vasee S. Moorthy, Johan Vekemans

**Affiliations:** a National Center for Immunization and Respiratory Diseases, Division of Bacterial Diseases, Centers for Disease Control and Prevention, Atlanta, GA 30329-4027, USA; b Epidemic Intelligence Service, Centers for Disease Control and Prevention, Atlanta, GA 30333, USA; c Center for Vaccine Innovation and Access, PATH, Seattle, WA 98121, USA; d Department of Science and Technology/National Research Foundation: Vaccine Preventable Diseases, and Medical Research Council: Respiratory and Meningeal Pathogens Research Unit, University of the Witwatersrand, Johannesburg, South Africa; e Department of Pediatrics, Molecular Virology and Microbiology, Baylor College of Medicine, Houston, TX 77030, USA; f Bill & Melinda Gates Foundation, Seattle, WA 98109, USA; g MRC Tropical Epidemiology Group, London School of Hygiene & Tropical Medicine, London, UK; h Initiative for Vaccine Research, World Health Organization, CH-1211 Geneva 27, Switzerland

**Keywords:** Group B *Streptococcus*, Maternal vaccination, Phase III trial, Vaccine licensure

## Abstract

Globally, group B *Streptococcus* (GBS) remains a leading cause of sepsis and meningitis in infants in the first 90 days of life. Intrapartum antibiotic prophylaxis (IAP) for women at increased risk of transmitting GBS to their newborns has been effective in reducing part, but not all, of the GBS disease burden in many high income countries (HICs). In low- and middle-income countries (LMICs), IAP use is low. Immunization of pregnant women with a GBS vaccine represents an alternative strategy to protecting newborns and young infants, through transplacental antibody transfer and potentially by reducing new vaginal colonization. This vaccination strategy was first suggested in the 1970s and several potential GBS vaccines have completed phase I/II clinical trials. During the 2015 WHO Product Development for Vaccines Advisory Committee meeting, GBS was identified as a high priority for the development of a vaccine for maternal immunization because of the major public health burden posed by GBS in LMICs, and the high technical feasibility for successful development. Following this meeting, the first WHO technical consultation on GBS vaccines was held on the 27th and 28th of April 2016, to consider development pathways for such vaccines, focused on their potential role in reducing newborn and young infant deaths and possibly stillbirths in LMICs. Discussion topics included: (1) pathophysiology of disease; (2) current gaps in the knowledge of global disease burden and serotype distribution; (3) vaccine candidates under development; (4) design considerations for phase III trials; and (5) pathways to licensure, policy recommendations and use. Efforts to address gaps identified in each of these areas are needed to establish the public health need for, the development and deployment of, efficacious GBS vaccines. In particular, more work is required to understand the global disease burden of GBS-associated stillbirths, and to develop quality-assured standardized antibody assays to identify correlates of protection.

## 1 Introduction and objectives

In 2014, the World Health Organization (WHO) convened the first meeting of the Product Development for Vaccines Advisory Committee (PDVAC). The committee was given the remit to review the status of vaccines under development, whose licensure and use may contribute importantly to reducing the disease burden in low- and middle-income countries (LMICs). At the meetings of the committee in 2014 and 2015 the status of vaccine development against 18 pathogens was reviewed [1]. At these meetings Respiratory Syncytial Virus (RSV) and group B *Streptococcus* (GBS) were identified as important pathogens causing a large burden of disease among neonates and infants in LMICs that may be amenable to prevention by immunization, including by maternal vaccination in pregnancy [1].

On the 27th and 28th of April 2016, WHO convened their first technical consultation on GBS vaccines, with participants drawn from academia, industry, public health agencies, funding bodies and regulatory authorities. Discussions focused on the development of GBS vaccines for maternal immunization, with emphasis on specific needs in LMICs. Topics discussed included: (1) pathophysiology of GBS disease; (2) current gaps in the knowledge of global GBS disease burden and serotype distribution; (3) vaccine products under development; (4) design considerations for phase III trials; and (5) pathways to licensure, policy recommendation and use.

## 2 GBS pathophysiology and disease syndromes, basic bacteriology

Neonatal and young infant GBS disease can be classified into early-onset disease (EOD, onset during the first 6 days of life), and late-onset disease (LOD, onset between days 7–89 of life). It is estimated that 60–90% of EOD occurs on the first day of life [2], [3]. GBS colonizes the human gastrointestinal and genitourinary tracts, and throat, and vertical transmission from colonized mothers can lead to invasive disease in their offspring. Disease in neonates and young infants develops as a result of invasion of GBS across epithelial cells into the bloodstream [4]. HIV-exposed infants are at a higher risk of developing invasive GBS disease [2], [5].

GBS has also been associated with stillbirths and prematurity, through mechanisms that remain poorly understood [6], [7]. Additionally, during pregnancy and postpartum, women are at increased risk of developing invasive GBS disease [8].

GBS produces a polysaccharide capsule of 10 antigenic types (Ia, Ib, II, III, IV, V, VI, VII, VIII, IX). In 1976, it was reported that transplacental transfer of maternal antibodies to type III capsular polysaccharide (CPS) was associated with protection against CPS type III GBS invasive disease in infants [9]. Results from subsequent studies supported this finding and generalized this to other GBS serotypes [10], [11], [12], [13], providing a rationale for maternal GBS vaccination targeting CPS to prevent disease in young infants.

Proteins such as alpha-C-protein (bca), C alpha-like proteins 2 and 3 (alp2 and alp3), epsilon/Alp1, Rib (rib), and beta-C-protein (bac) are embedded in the GBS bacterial surface, and are also candidate vaccine targets.

## 3 GBS disease management and prevention practices

WHO currently recommends intrapartum antibiotic prophylaxis (IAP) administered intravenously for women with GBS colonization to prevent early neonatal GBS infection, but acknowledges that systematic GBS screening may not be feasible in many settings, and the presence of other risk factors should be considered [14]. IAP is recommended for women with preterm pre-labour rupture of membranes, but not for women in preterm labour with intact amniotic membranes, nor for women with pre-labour rupture of membranes at term or near term (36 weeks gestation and above). The latter is based on evidence from studies including women with membrane rupture duration under 12 h, and it is acknowledged that there may be a benefit from IAP in women with prolonged rupture of membranes (>18 h) [14].

High income countries that have implemented IAP select pregnant women for treatment by either screening for GBS colonization or by monitoring them for known intrapartum risk factors. South Africa, an upper-middle income country, has introduced a risk-based IAP strategy, but with limited uptake [15]. A multiplicity of access to care and health system challenges complicate the implementation of IAP in many LMIC settings.

For neonates and young infants, WHO recommends empirical treatment with ampicillin and gentamicin at birth for all high-risk neonates (membranes ruptured > 18 h before delivery, maternal fever before delivery and during labour, foul-smelling or purulent amniotic fluid) [16]. However, many countries, particularly those in sub-Saharan Africa, experience challenges in implementing these recommendations.

WHO currently recommends that pregnant women have at least four antenatal visits to a health care facility, but in many LMICs the actual number of visits is less [17]. Nevertheless, more than 80% of pregnant women in sub-Saharan Africa make at least one antenatal care visit, providing an opportunity for administering maternal vaccination, if the visits occur with sufficient time prior to delivery for a protective immune response to develop. That said, the proportion of women making at least one antenatal visit varies widely between countries (e.g., in 2014 from 41% in Ethiopia to 99% in Swaziland) [18].

## 4 Global GBS disease burden

In a global meta-analysis, countries in the WHO Africa region had the highest pooled estimate of young infant (day 0–89) invasive GBS disease (pooled incidence 1.21/1000 livebirths, 95% CI: 0.50–1.91) [19]. In a more recent systematic review, focusing on data from sub-Saharan African countries, it was estimated that the incidence may be twice as high [20]. The lowest pooled estimate in the global review was reported for Southeast Asia (pooled incidence 0.02/1000 livebirths, 95% CI: 0.00–0.07) [19]. It is unclear why the burden of GBS disease appears to be low in this region and more rigorous studies in Southeast Asia are needed to determine if this is due to under-ascertainment or to true differences in the incidence of GBS disease in this region.

In an analysis of the global distribution of GBS, five serotypes (Ia, Ib, II, III, V) accounted for 94% of invasive disease in young infants [19]. A limitation of this analysis is that there were no serotype data reported from Southeast Asia and only two studies from the African region. Recent reports from Kenya and The Gambia showed that these five serotypes were also the most frequent [21], [22]. Data from Central Africa are sparse. Limited serotype data from Southeast Asia suggest that there might be variations in serotype distribution within regions [23]. The CC17 hypervirulent clone has classically been restricted to the type III serotype but capsular switching and possible emergence of a hypervirulent type IV serotype has been reported [24].

Data on the global burden of GBS-related stillbirths are sparse. In a recent systematic review, the proportion of stillbirths associated with GBS infection ranged from 0 to 12%. However, the paucity of data and lack of standardized case definitions for stillbirths limited the conclusions that could be drawn [25]. Two recent, rigorously conducted prospective studies of stillbirths in Kenya [21] and South Africa [Madhi et al., in progress] found that the incidence of GBS-associated stillbirths was similar to or exceeded that of invasive EOD in their respective settings. These studies, which included culture of GBS from either cord blood or blood obtained from heart-puncture, suggest that GBS associated stillbirth might be part of the continuum of the clinical spectrum of EOD, in which the majority of cases present at birth.

Accurate estimates of the GBS disease burden in LMICs are difficult to obtain for a number of reasons, including: (1) failure to obtain specimens in high neonatal mortality settings, particularly immediately after and within the first 24 h of birth when most EOD occurs; (2) limited GBS recovery from specimens that are collected and processed through suboptimal methods; (3) the frequent initiation of antibiotic therapy before specimen collection and (4) absence of denominator data for incidence estimates. Unfortunately, regions with the highest burden of neonatal deaths are those most impacted by these problems.

Data about young infant GBS disease in high income countries (HICs) further support the need for a vaccine. The United States (US) recommends universal screening and IAP in all colonized women. The first consensus guideline for the prevention of perinatal invasive GBS disease was issued in 1996, resulting in a greater than 80% reduction in EOD [26]. However, IAP did not lead to reductions in the incidence of LOD [26], and there are still approximately 2000 cases annually of young infant invasive GBS disease in the US. Moreover, over 30% of neonates delivered in the US are exposed to intrapartum antibiotics, raising concerns about the effects of such use on antimicrobial resistance and the potential impact on the newborn’s microbiome. European countries have adopted a range of screening and risk-based approaches to GBS prevention. Despite these, GBS remains the leading cause of bacterial meningitis in young infants [27], with significant mortality and long-term morbidity. Recently some countries, including the Netherlands [28] and the United Kingdom [29], have reported increases in both EOD and LOD. Data from both the US and Europe indicate that the distribution of serotypes causing young infant disease has been relatively stable over time, with over 90% of cases attributable to serotypes Ia, Ib, II, III, and V [30], [31], [32].

## 5 GBS vaccine candidates

Currently, CPS conjugate vaccines and protein-based GBS vaccines are under development. The conjugate vaccines use GBS CPS as the primary target and enhance immunogenicity by covalent conjugation of a protein carrier, such as tetanus toxoid or CRM_197_ (a non-toxic mutant of diphtheria toxin). Development of these products builds on a history of successfully licensed conjugate vaccines for *Haemophilus influenzae* type b (Hib), *Neisseria meningitidis*, and *Streptococcus pneumoniae*, shown to protect vaccinated individuals against disease and to prevent acquisition of bacterial carriage, leading to herd immunity. Some limitations of multivalent conjugate vaccines include their complex manufacturing process, reflected in their pricing, and the fact that they confer protection only against included serotypes. Protein vaccines use common GBS protein(s) as the vaccine target, and have the potential to confer broad protection across serotypes. In addition, they may be less complex and less expensive to manufacture. However, the relationship between antibodies against surface proteins and development of disease has not been established.

Maternal vaccination of mice followed by challenge of neonatal pups has frequently been used to evaluate GBS vaccine candidates [33], [34]. Such models can shed light on the functional activity of immunogens, such as specific protein antigen targets [35], [36]. They can also contribute to understanding of disease pathophysiology and study of factors leading from colonization to invasion [37], [38], [39]. Animal reproductive toxicology studies are typically required before evaluation in pregnant women.

GlaxoSmithKline (GSK) (previously Novartis) has sponsored phase I and II trials of an investigational trivalent (Ia, Ib, III) CPS-CRM_197_ GBS conjugate vaccine, and are currently pursuing pre-clinical studies of a pentavalent (Ia, Ib, II, III, V) CPS-CRM_197_ vaccine. Their vaccine is being developed for immunization in pregnancy to prevent subsequent invasive GBS disease in neonates and young infants. A total of 1732 non-pregnant women and 610 pregnant women have been vaccinated with the trivalent vaccine in phase I/II trials. The vaccine was well-tolerated, with no safety signals of concern in either mothers or infants. Reported local and systematic reactions were mostly mild to moderate, and no vaccine-related serious adverse events were reported. Over 75% of both non-pregnant and pregnant vaccine recipients demonstrated a more than four-fold rise in serotype-specific IgG concentrations, and mother to infant IgG transfer rates were 50–81% across all serotypes, similar to that observed with other polysaccharide conjugate vaccines [40], [41], [42], [43]. CPS-specific antibody concentrations in infants of vaccinated mothers remained, for at least 90 days, above that of infants whose mothers were administered placebo. There was no evidence of an enhanced immune response with two vaccine doses compared to one, or with the use of aluminum hydroxide adjuvant [41], or of interference with responses to infant diphtheria vaccination [40]. However, vaccine responses were lower among women with no detectable anti-GBS IgG at baseline and among HIV-infected women [43].

Pfizer is also in early phase development with a candidate CPS-CRM_197_ vaccine for the prevention of GBS invasive disease in infants, through maternal immunization during pregnancy. This vaccine is being developed using the platform developed for other conjugate vaccines (e.g., 13-valent pneumococcal conjugate vaccine).

MinervaX is investigating a protein-based vaccine candidate, based upon a fusion protein of the N-terminal domains of Alpha-like proteins, Rib and AlpC (GBS-NN). Alpha-like proteins are a family of GBS surface proteins which are found on almost all GBS isolates analyzed to date. Preliminary findings in a case-control epidemiologic study indicated that antibodies against full-length AlpC and Rib transfer efficiently across the placenta, and low antibody concentrations to AlpC and Rib in neonates correlated to increased susceptibility to invasive GBS infection caused by strains expressing Rib [44]. Minervax has completed phase Ia studies with GBS-NN in non-pregnant female volunteers, and a phase Ib study is underway among non-pregnant women to test for safety and dose-confirmation. Results to date have shown favourable safety and immunogenicity of GBS-NN in non-pregnant women. The current strategy is based on a one- or two-dose vaccine schedule with aluminum hydroxide adjuvant, for administration early in the third trimester of pregnancy. Further studies on correlates of protection and maternal transfer of naturally occurring GBS-NN antibodies are ongoing.

## 6 GBS vaccine development considerations for LMICs

There are several obstacles to research on, and ultimately deployment of, GBS vaccines in LMICs, including infrastructure challenges, limited financial resources, health systems deficiencies, and limited regulatory experience for product licensure. However, public-private partnership initiatives and innovative financing mechanisms can help to overcome these and several capacity strengthening initiatives are ongoing. PATH’s Center for Vaccine Innovation and Access aims to accelerate the development of vaccines that will be effective and affordable in the countries that most urgently need them [45]. Their GBS vaccine programme focuses on working with developing country vaccine manufacturers to develop a polyvalent GBS conjugate vaccine.

The only published analysis of the economic impact of implementing GBS vaccines in LMICs is by Kim and colleagues for South Africa [46]. With the assumption of 10–30 USD/dose of vaccine (which is well above the classical GAVI procurement price range), vaccine efficacy against included serotypes of 50–90% among term infants, with lower efficacy among preterm infants, and vaccine coverage of 75%, maternal immunization was assessed to be very cost effective (range 416–3545 USD/DALY averted). A cost-effectiveness analysis for GAVI-eligible countries in sub-Saharan Africa is currently being conducted by Sinha and colleagues. There is a need for further work on the impact and cost-effectiveness of candidate GBS vaccines, especially focusing on low income settings, taking into account recent data on the role of GBS on stillbirths. Cost-effectiveness models for HICs need to be updated taking into account current disease trends.

## 7 Regulatory considerations

While, to our knowledge, no vaccine has so far obtained regulatory approval for labeling for use during pregnancy, several vaccines are recommended for use during pregnancy by public health authorities, and progress is being made in the regulatory pathway to vaccine licensure for use in pregnancy [47]. Clinical development can be overseen in the United States by the investigational new drug (IND) programme, possibly followed by a Biological License Application. In Europe, a Marketing Authorisation request can be submitted to the European Medicine Agency (EMA). The EMA can also conduct a regulatory review for products intended for use outside Europe through the Article 58 pathway. Submissions for registration can be made as specified by relevant national regulatory authorities in LMICs. For sub-Saharan African countries, the African Vaccine Regulatory Forum (AVAREF) has played a key role in facilitating timely regulatory authorization and approvals of group A meningococcal conjugate vaccine (MenAfriVac®) through its joint reviews. AVAREF also has a track record of facilitating clinical trial authorization at the pre-licensure stage [48]. The Developing Country Vaccine Regulators’ Network (DCVRN) may also facilitate steps in regulatory processes in LMICs [49].

Studies to demonstrate the clinical benefit of new vaccines are generally required by regulators, either pre- or post-licensure. Pivotal data to support licensure could be derived from a randomized, double-blind placebo-controlled trial, with a relevant GBS disease entity as primary endpoint. Regulators may consider licensure based on serological endpoints if determined to be reasonably likely to predict clinical benefit. In the case of the FDA, this may lead to an accelerated approval pathway, which requires post-licensure studies to verify and describe clinical benefit [50]. Irrespective of the licensure pathway, policy decision and the adoption of a vaccine into a public health programme will likely require demonstration of both safety and clinical benefit.

Adequate safety profile characterization is paramount. Standard case definitions for outcomes of relevance to maternal immunization including infant outcomes will be important. In this context, recent efforts by the Global Alignment of Immunization Safety Assessment in pregnancy (GAIA) working group coordinated by the Brighton Collaboration Foundation should prove useful [51].

Pre-licensure studies will likely be required to demonstrate that maternal immunization does not adversely interfere with an infant’s response to other relevant vaccines, due to the transfer of maternal antibodies to the carrier proteins. Co-administration studies with other vaccines recommended for maternal immunization may also be important.

## 8 Considerations for phase III trials

A phase III trial with a primary clinical disease endpoint would provide the most compelling evidence of efficacy, and should include a nested immunogenicity study to allow for evaluation of correlates of protection.

Culture-confirmed invasive GBS disease in young infants would likely meet regulatory requirements for a primary endpoint. This is a clinically relevant endpoint of public health relevance that can be defined with high specificity. Similar clinical endpoints have been used in trials to support the licensure of other conjugate vaccines. Since blood culture positivity rates vary according to the clinical criteria guiding sample collection, as well as sample quantity, quality and processing methods (e.g., manual vs. automated blood culture), standardization of these procedures across trial sites will be needed. Use of molecular tests, such as PCR, might help increase sensitivity, but further data are needed to assess the impact on specificity of the endpoint case definition. Because GBS-associated stillbirths can be considered as a continuum of neonatal GBS disease due to their similar pathophysiology, consideration should be given to the inclusion of GBS-confirmed stillbirths with young infant disease as part of a composite disease endpoint. Two recent studies in Kenya [21] and South Africa [Madhi et al., in progress] that collected sterile site samples from stillbirths for identification of GBS may provide a foundation for the development of standardized case definitions for GBS-associated stillbirths.

A key issue for GBS vaccines is the possibly low baseline incidence of the primary endpoint of invasive disease, requiring phase III clinical efficacy trials to be very large. For example, in a population with a baseline incidence of GBS invasive disease of 2.0 per 1000 live births, assuming a proportion of cases are not eligible for endpoint inclusion and assuming a 75% vaccine efficacy, a trial size of 40,000–60,000 mother-baby dyads would be required [52]. Such high sample size requirements would pose both logistic and cost issues. Having a composite disease endpoint, for example including both GBS-related stillbirth and invasive GBS disease in neonates and young infants, would reduce the required trial size.

Other considerations in trial design and trial site selection include: the availability of reliable baseline incidence data; prevalence of underlying conditions that could affect the immune response to vaccination, such as HIV; home-delivery rates (considering that most EOD occurs during the first 24 h of life); diagnostic capacity for both safety and efficacy evaluation; capacity to assess sepsis and gestational age; barriers to access to care; Good Clinical Practice research experience; and national regulatory and local ethical review capacity.

In settings with very low baseline GBS-related disease incidence in the context of optimal preventive care, conducting an efficacy trial of a GBS vaccine may not be feasible. However, in high GBS burden settings where universal screening during pregnancy is not the local standard of care and IAP is not effectively implemented, a placebo-controlled vaccine trial, without introducing screening-based IAP, could be considered ethically acceptable [53]. Lack of post-trial sustainability, the possible creation of a double standard of care in the same community and the possibility of undue inducement to study participation were seen as arguments against the specific implementation of screening-based IAP in a phase III trial in many LMIC settings. Locally-approved criteria for antibiotic treatment initiation should be defined in standard operating procedures, delineating essential needs for antenatal care, delivery, and postnatal care, following national guidelines when available, considering WHO recommendations and the local context. Samples for bacterial cultures from potential cases should be obtained before antibiotic administration, which should be documented, in line with the 2013 WHO recommendation for postnatal empiric antibiotics for high-risk infants [16].

It is critical that host countries and trial communities are appropriately engaged in the planning and conduct of pre-licensure trials and in vaccine implementation plans after successful licensure and recommendations for use. Trials should be conducted only in settings where there is an intent to introduce a vaccine shown to be efficacious.

The characterization of a correlate of protection would be of high-value, especially for bridging studies in different population groups. It is unclear the extent to which correlates of protection may be inferred from the evaluation of natural immunity in observational studies. In this respect, it will be important to assess the equivalence between GBS antigen-specific IgG concentrations and functional antibody levels in naturally immunized versus vaccinated subjects. In addition, correlates of protection may vary by serotype and by disease entity.

There is an urgent need for standardized assays, to support between-study comparisons and bridging studies, with regulatory-acceptable quality systems, both for serological antibody binding assays such as enzyme-linked immunosorbent assays (ELISA) and functional assays such as opsonophagocytic activity (OPA) assays that measure bacterial killing. GBS assay standardization initiatives are ongoing. Keys to success in standardizing pneumococcal assays included: (1) involvement of manufacturers and regulators from the outset; (2) willingness of laboratories to adjust their protocols; (3) availability of a central source of relevant reagents, such as reference sera, antigen sources, bacterial strains; and (4) established WHO reference laboratories as part of WHO’s Collaborating Centre networks. It will be very helpful to include laboratories with previous experience working with WHO, and regulators and manufacturers with previous experience of conjugate vaccine approaches (bearing in mind, also, possible protein approaches to vaccine development). Pfizer and GSK are exploring the possibility to supply unconjugated GBS CPS to a GBS consortium through a third party, such as the UK National Institute for Biological Standards and Control.

Maternal or newborn colonization may potentially serve as a secondary endpoint in phase III trials, given that newborn GBS colonization or exposure from colonized mothers is a precursor to young infant invasive GBS disease. An early serotype III conjugate vaccine candidate tested in non-pregnant women indeed showed reduced rates of acquisition of type-specific GBS colonization [54]. Colonization was not viewed, however, as a potential licensure endpoint based on the view that efficacy against colonization may not necessarily reflect efficacy against disease, as virulence and other factors may influence whether colonization leads to invasive disease, and circulating antibodies may prevent invasive disease but not colonization [55].

There was general agreement that it would not be reasonable to expect that a phase III study be powered to produce CPS serotype-specific efficacy estimates. Post-licensure evaluations will play a critical role in characterizing rarer safety events than are detectable in a phase III trial and effectiveness under “real-world” conditions, as well as in special populations of interest or for obtaining serotype-specific results. Post-licensure monitoring should include surveillance for capsular switching and serotype replacement, as has been observed with pneumococcus. The safety requirements and possibility of emergence of escape variants should also be considered for protein vaccines, given that there are sequence polymorphisms.

## 9 Gaps and next steps

In September 2015, PDVAC recommended that WHO develop guidance on development pathways for GBS vaccines [1]. While the April 2016 GBS consultation convened by WHO was an important step, the need, as PDVAC highlighted, for consensus on strategic goals, trial design considerations and development of preferred product characteristics remains critical and should be addressed in the near term. WHO will progress towards the publication of a GBS vaccine development roadmap. Work to address several key knowledge gaps (Table 1), particularly efforts underway to update global disease burden estimates, and to standardize immunological assays, will maintain momentum and facilitate preparation for phase III trials. Participants highlighted several additional areas for research that can be of value in parallel with product development, including: assessments of pregnant women and healthcare worker attitudes and concerns regarding maternal immunization in LMICs; efforts to raise awareness of the burden of GBS disease and to sensitize the public health community about the potential value of a GBS vaccine, particularly in countries that may lack local data on the GBS burden; synthesizing lessons from experience with maternal immunization in LMICs against influenza, tetanus and pertussis and identifying any synergies with newly launching efforts related to maternal RSV immunization; and identifying likely routes for maternal immunization financing for LMIC, particularly as more countries in sub-Saharan Africa and Southeast Asia transition from eligibility for GAVI financing to partial or full self-financing.

**Table 1:** Knowledge gaps, areas for further research.

Topic	Knowledge gaps, areas for research
GBS pathophysiology	Pathophysiology of GBS-associated disease and conditions
	• Factors determining progression from colonization to LOD • Progression from maternal colonization to ascending infection and stillbirth • Relationship between maternal colonization and preterm births • Differential risk factors for LOD compared to EOD
GBS epidemiology in LMICs	Young infant disease
	• Preterm contribution to young infant disease burden, particularly in LMICs where preterm survival may be lower • LOD epidemiology in LMICs ∘ Understanding differences from that described in HICs (e.g., proportion preterm, ratio of early to late-onset disease, median age of onset, role of maternal HIV infection) • Role of socio-economic status, urbanization, and birth location (facility type, home vs facility) in GBS disease risk
	GBS-associated stillbirth
	• Risk factors associated with GBS-associated stillbirth
GBS disease burden	Surveillance challenges
	• Lack of specimens from ill newborns in high neonatal mortality settings • Failure to obtain specimens in the first days of life • Limited sensitivity of cultures used to detect disease • Difficulty in isolating and characterizing GBS from colonized specimens • Collection of denominator data for incidence estimates
	Young infant disease burden
	• More information on burden from LMICs, especially South Asia • Sub-Saharan Africa: representation beyond southern Africa ∘ Areas with low maternal HIV ∘ Low-income countries (LICs) • More evidence on disease burden from other high mortality regions • More comprehensive serotype and protein type diversity data from LMICs, especially LICs
	Pregnancy-associated disease burden
	• Limited information from LMICs
	GBS-associated stillbirth burden
	• Need for a standardized definition • Challenges in obtaining appropriate specimens
	Non-pregnant adults
	• Limited information from LMICs
GBS disease management and prevention practices	Knowledge gaps related to perinatal care in LMIC
	• Current status of ANC visit uptake in LMICs (as a potential maternal vaccine delivery channel) • Status of implementation of WHO-recommended perinatal care in LMICs • Understanding the challenges associated with IAP implementation in LMICs
Getting a product to phase III	Universal gaps
	• Reproductive toxicity animal model validation • Assay standardization • Establishing correlates of protection • Ascertaining appropriate window during pregnancy for vaccination to achieve optimal antibody transfer to the newborn • Assessment of immune interference with other maternal and infant vaccines
	Conjugate vaccines
	• Immunogenicity and safety data on higher-valency candidate vaccines • Ascertainment of whether a single dose elicits adequate immune response, especially in women without detectable baseline antibody and in women with HIV infection
	Protein vaccines
	• Safety and immunogenicity data in pregnant women • Assessment of whether an adequate immune response can be achieved by a single-dose and whether adjuvant is required • Foundational work on correlates of protection and appropriate immunogenicity assays pending
GBS vaccine development considerations for LMICs	• Strengthen dual vaccine development pathway (for use in both HICs and LMICs) • Need for a mechanism to support vaccine development in LMICs ∘ Supporting LMIC manufacturers ∘ Supporting local regulatory authorities • Limited assessment on cost-effectiveness for LICs (one for sub-Saharan Africa in progress)
Regulatory considerations	Acceptable licensure pathways
	• Clarification of conditions where a substitute endpoint for clinical disease (for example an immune correlate of protection) may be acceptable for licensure • Clarification of pathways for generalizing results across the different settings where vaccine may be used
Planning for phase III trials	Consensus building
	• GBS maternal immunization strategic objectives • Preferred product characteristics • Licensure trial design considerations, including case definitions and disease endpoints • Flexible clinical development pathway options, depending on generated evidence
	Study site preparation
	• Study site selection • Development of study standard operating procedures and capacity for trial implementation
Preparing for post-licensure needs to facilitate implementation	Building stakeholder commitment
	• Characterize concerns regarding maternal immunization among pregnant women and healthcare workers • Increase awareness about GBS disease • Sensitize the public health community about the potential value of a GBS vaccine
	Minimizing the evidence gap for implementation
	• Advanced planning to support a SAGE policy recommendation and WHO prequalification

Beyond licensure considerations, it is also important to consider, at an early stage, strategies to reduce the time between licensure and vaccine deployment in LMICs [56]. Support from United Nations procurement agencies and GAVI funding require both WHO prequalification and policy recommendation (through WHO Strategic Advisory Group of Experts (SAGE) on Immunization) [57]. Such policy recommendations depend on additional factors beyond those considered during regulatory review, including implementation feasibility, impact on health systems, integration with other interventions and cost-effectiveness. Advance planning for provision of key evidence may help shorten the interval between regulatory approval and implementation scale-up [58].

Successful licensure and implementation of RSV vaccines, which are at a more advanced stage of development, may help fill key implementation evidence gaps and strengthen the maternal immunization platform, laying the groundwork for a rapid rollout of a GBS maternal immunization programme once a safe and effective vaccine is approved by national regulatory authorities and recommended for use by policy makers.

## Meeting attendees

Participants: Mark Alderson (PATH, Seattle, USA), Carol J. Baker (Baylor College of Medicine, Houston, USA), Azucena Bardaji (Manhica Health Research Centre, Mozambique and ISGlobal, Barcelona, Spain), Hellen Cherono Barsosio (KEMRI-Wellcome Trust Research Programme, Kilifi, Kenya), Jay Berkley (KEMRI-Wellcome Trust Research Programme, Kilifi, Kenya), Adriane de Oliveira (Agência Nacional de Vigilância Sanitária, Brasilia, Brazil), Andrew Gorringe (Public Health England, Porton Down, UK), Ruth Gilbert (Institute of Child Health, London, UK), David Goldblatt (University College London Medical School, London, UK), Paul T. Heath (Vaccine Institute, St Georges, University of London, London, UK), Philip Henneke (Center for Pediatrics and Center for Chronic Immunodeficiency, University Medical Centre, Freiburg, Germany), Margaret Ip (Chinese University of Hong Kong, Hong Kong, China), Beate Kampmann (MRC, Banjul, Gambia), Eric Karikari-Boateng (Food and Drugs Board Ghana, Accra, Ghana), David Kaslow, PATH, Seattle, USA), John Kinuthia (Kenyatta National Hospital, Kilifi, Kenya), Miwako Kobayashi (Centers for Disease Control and Prevention, Atlanta, USA), Gaurav Kwatra (WITS University, Johannesburg, South Africa), Bengt Johansson Lindbom (Lund University, Sweden), Shabir A. Madhi (National Institute for Communicable Diseases, Johannesburg, South Africa), Fatme Mawas (NIBSC, MHRA, Potters Bar, UK), Kirsty Mehring-Le Doare (Imperial College Faculty of Medicine, London, UK), Malcolm Molyneux (Liverpool School of Tropical Medicine, Liverpool, UK), Moon Nahm (University of Alabama at Birmingham, Birmingham, USA), Patricia Njuguna (KEMRI-Wellcome Trust Research Programme, Kilifi, Kenya), Steven K. Obaro (University of Nebraska Medical Center, Omaha Nebraska, USA), Eric Pelfrene (European Medicines Agency, London, UK), Fernando Polack (Fundación INFANT, Buenos Aires, Argentina), Adam Ratner (New York University School of Medicine, New York, USA), Laura Riley (Massachusetts General Hospital, Boston, USA), Jeff Roberts (Food and Drug Administration, Silver Spring, USA), Samir Saha (Institute of Child Health, Dhaka, Bangladesh), Stephanie Schrag (Centre for Disease Control and Prevention, Atlanta, USA), Anna Seale (London School of Hygiene and Tropical Medicine, London, UK), Betuel Sigaúque (Centro de Investigação em Saúde de Manhiça Maputo, Mozambique), Peter Smith (London School of Hygiene and Tropical Medicine, London, UK), Igor Smolenov (PATH, Seattle, USA), Ajoke Sobanjo-Ter Meulen (Bill & Melinda Gates Foundation, Seattle, USA), Andi Tate (PATH, Seattle, USA), Amina White (University of North Carolina, Chapel Hill, USA).

Invited industry representative: Analisa S. Anderson (Pfizer Vaccine Research and Development, Pearl River NY, USA), Muriel Pasté (Pfizer, Brussels, Belgium), Ilse Dieussaert (GlaxoSmithKline Vaccines, Wavre, Belgium), Per Fischer (MinervaX ApS, Copenhagen, Denmark), Ouzama Henry (GlaxoSmithKline Vaccines, Wavre, Belgium), Immaculada Margarit Ros (GlaxoSmithKline Vaccines, Sienna, Italy), Patrick Tippoo, The Biovac Institute, Pinelands, South Africa), Wendy Watson (Pfizer, Collegeville PA, USA), Seanette Wilson (The Biovac Institute, Pinelands, South Africa).

WHO secretariat: Brigitte Giersing (Initiative for Vaccine Research, WHO, Geneva, Switzerland), Joachim Hombach (Initiative for Vaccine Research, WHO, Geneva, Switzerland), Ivana Knezevic (Technologies Standards and Norms, WHO, Geneva, Switzerland), Vasee Moorthy (Initiative for Vaccine Research, WHO, Geneva, Switzerland), Johan Vekemans (Initiative for Vaccine Research WHO, Geneva, Switzerland), Mike Ward (Regulatory Systems Strengthening, HIS/RSS, WHO HQ, Geneva, Switzerland).

## Conflict of interest statements

Dr. Shabir Mahdi’s institution received grant support from Novartis/GSK, Pfizer and Minervax related to GBS epidemiology and/or immunology studies. Dr. Shabir Mahdi and Dr. Carol Baker received honoraria from Pfizer for scientific advice.

## Funding

This work was supported by the Bill & Melinda Gates Foundation, Seattle, WA [Global Health Grant OPP1134011].

## References

[1] Giersing B.K., Modjarrad K., Kaslow D.C., Moorthy V.S. (2016). Report from the World Health Organization's Product Development for Vaccines Advisory Committee (PDVAC) meeting, Geneva, 7–9th Sep 2015. Vaccine.

[2] Cutland C.L., Schrag S.J., Thigpen M.C., Velaphi S.C., Wadula J., Adrian P.V. (2015). Increased risk for group B *Streptococcus sepsis* in young infants exposed to HIV, Soweto, South Africa, 2004–2008(1). Emerg Infect Dis.

[3] Schuchat A. (1998). Epidemiology of group B streptococcal disease in the United States: shifting paradigms. Clin Microbiol Rev.

[4] Edwards M.S., Nizet V., Baker C.J., Wilson C.B., Nizet V., Maldonado Y.A., Remington J.S., Klein J.O. (2016). Group B streptococcal infections. Remington and Klein's infectious diseases of the fetus and newborn infant.

[5] Epalza C., Goetghebuer T., Hainaut M., Prayez F., Barlow P., Dediste A. (2010). High incidence of invasive group B streptococcal infections in HIV-exposed uninfected infants. Pediatrics.

[6] Goldenberg R.L., Thompson C. (2003). The infectious origins of stillbirth. Am J Obstet Gynecol.

[7] Turrentine M.A., Ramirez M.M. (2008). Recurrence of group B streptococci colonization in subsequent pregnancy. Obstet Gynecol.

[8] Deutscher M., Lewis M., Zell E.R., Taylor T.H., Van Beneden C., Schrag S. (2011). Incidence and severity of invasive *Streptococcus pneumoniae*, group A *Streptococcus*, and group B *Streptococcus* infections among pregnant and postpartum women. Clin Infect Dis: Off Publ Infect Dis Soc Am.

[9] Baker C.J., Kasper D.L. (1976). Correlation of maternal antibody deficiency with susceptibility to neonatal group B streptococcal infection. N Engl J Med.

[10] Dangor Z., Kwatra G., Izu A., Adrian P., Cutland C.L., Velaphi S. (2015). Correlates of protection of serotype-specific capsular antibody and invasive group B *Streptococcus* disease in South African infants. Vaccine.

[11] Baker C.J., Carey V.J., Rench M.A., Edwards M.S., Hillier S.L., Kasper D.L. (2014). Maternal antibody at delivery protects neonates from early onset group B streptococcal disease. J Infect Dis.

[12] Lin F.Y., Philips J.B., Azimi P.H., Weisman L.E., Clark P., Rhoads G.G. (2001). Level of maternal antibody required to protect neonates against early-onset disease caused by group B *Streptococcus* type Ia: a multicenter, seroepidemiology study. J Infect Dis.

[13] Lin F.Y., Weisman L.E., Azimi P.H., Philips J.B., Clark P., Regan J. (2004). Level of maternal IgG anti-group B *Streptococcus* type III antibody correlated with protection of neonates against early-onset disease caused by this pathogen. J Infect Dis.

[14] World Health Organization. WHO recommendations for prevention and treatment of maternal peripartum infections. Geneva, Switzerland; 2015. 26598777

[15] Schrag S.J., Cutland C.L., Zell E.R., Kuwanda L., Buchmann E.J., Velaphi S.C. (2012). Risk factors for neonatal sepsis and perinatal death among infants enrolled in the prevention of perinatal sepsis trial, Soweto, South Africa. Pediatr Infect Dis J.

[16] World Health Organization. Pocket book of hospital care for children: guidelines for the management of common childhood illness, 2nd ed.; 2013. 24006557

[17] World Health Organization. Maternal mortality; 2015.

[18] UNICEF. Antenatal care coverage. February 2016 ed; 2016.

[19] Edmond K.M., Kortsalioudaki C., Scott S., Schrag S.J., Zaidi A.K., Cousens S. (2012). Group B streptococcal disease in infants aged younger than 3 months: systematic review and meta-analysis. Lancet (London, England).

[20] Sinha A., Russell L.B., Tomczyk S., Verani J.R., Schrag S.J., Berkley J.A. (2016). Disease burden of group B *Streptococcus* among infants in sub-Saharan Africa: a systematic literature review and meta-analysis. Pediatr Infect Dis J.

[21] Seale A.C., Koech A.C., Sheppard A.E., Barsosio H.C., Langat J., Anyango E. (2016). Maternal colonization with *Streptococcus agalactiae* and associated stillbirth and neonatal disease in coastal Kenya. Nat Microbiol.

[22] Le Doare K., Jarju S., Darboe S., Warburton F., Gorringe A., Heath P.T. (2016). Risk factors for group B *Streptococcus* colonisation and disease in Gambian women and their infants. J Infect.

[23] Islam M.S., Saha S.K., Islam M., Modak J.K., Shah R., Talukder R.R. (2016). Prevalence, serotype distribution, and mortality risk associated with group B *Streptococcus* colonization of newborns in rural Bangladesh. Pediatr Infect Dis J.

[24] Bellais S., Six A., Fouet A., Longo M., Dmytruk N., Glaser P. (2012). Capsular switching in group B *Streptococcus* CC17 hypervirulent clone: a future challenge for polysaccharide vaccine development. J Infect Dis.

[25] Nan C., Dangor Z., Cutland C.L., Edwards M.S., Madhi S.A., Cunnington M.C. (2015). Maternal group B *Streptococcus*-related stillbirth: a systematic review. BJOG: An Int J Obstet Gynaecol.

[26] Schrag S.J., Verani J.R. (2013). Intrapartum antibiotic prophylaxis for the prevention of perinatal group B streptococcal disease: experience in the United States and implications for a potential group B streptococcal vaccine. Vaccine.

[27] Okike I.O., Johnson A.P., Henderson K.L., Blackburn R.M., Muller-Pebody B., Ladhani S.N. (2014). Incidence, etiology, and outcome of bacterial meningitis in infants aged <90 days in the United Kingdom and Republic of Ireland: prospective, enhanced, national population-based surveillance. Clin Infect Dis: Off Publ Infect Dis Soc Am.

[28] Bekker V., Bijlsma M.W., van de Beek D., Kuijpers T.W., van der Ende A. (2014). Incidence of invasive group B streptococcal disease and pathogen genotype distribution in newborn babies in the Netherlands over 25 years: a nationwide surveillance study. Lancet Infect Dis.

[29] O'Sullivan C., Lamagni T., Patel D., Efstratiou A., Reynolds A., Cunney R. (2016). Group B Streptococcal (GBS) disease in UK ad Irish infants younger than 90 days of age in 2014–15. Paper presented at the Royal College of Paediatrics and Child Health, 26–28 April, 2016, Liverpool, UK Abstract published. Arch Dis Child.

[30] Melin P., Efstratiou A. (2013). Group B streptococcal epidemiology and vaccine needs in developed countries. Vaccine.

[31] Jordan H.T., Farley M.M., Craig A., Mohle-Boetani J., Harrison L.H., Petit S. (2008). Revisiting the need for vaccine prevention of late-onset neonatal group B streptococcal disease: a multistate, population-based analysis. Pediatr Infect Dis J.

[32] Phares C.R., Lynfield R., Farley M.M., Mohle-Boetani J., Harrison L.H., Petit S. (2008). Epidemiology of invasive group B streptococcal disease in the United States, 1999–2005. JAMA.

[33] Paoletti L.C., Madoff L.C. (2002). Vaccines to prevent neonatal GBS infection. Semin Neonatol: SN.

[34] Paoletti L.C., Pinel J., Kennedy R.C., Kasper D.L. (2000). Maternal antibody transfer in baboons and mice vaccinated with a group B streptococcal polysaccharide conjugate. J Infect Dis.

[35] Larsson C., Stalhammar-Carlemalm M., Lindahl G. (1999). Protection against experimental infection with group B *Streptococcus* by immunization with a bivalent protein vaccine. Vaccine.

[36] Gravekamp C., Kasper D.L., Michel J.L., Kling D.E., Carey V., Madoff L.C. (1997). Immunogenicity and protective efficacy of the alpha C protein of group B streptococci are inversely related to the number of repeats. Infect Immun.

[37] Randis T.M., Gelber S.E., Hooven T.A., Abellar R.G., Akabas L.H., Lewis E.L. (2014). Group B *Streptococcus* beta-hemolysin/cytolysin breaches maternal-fetal barriers to cause preterm birth and intrauterine fetal demise in vivo. J Infect Dis.

[38] Ancona R.J., Ferrieri P. (1979). Experimental vaginal colonization and mother-infant transmission of group B streptococci in rats. Infect Immun.

[39] McDuffie R.S., Gibbs R.S. (1996). Ascending group B streptococcal genital infection in the rabbit model. Am J Obstet Gynecol.

[40] Donders G.G., Halperin S.A., Devlieger R., Baker S., Forte P., Wittke F. (2016). Maternal immunization with an investigational trivalent group b streptococcal vaccine: a randomized controlled trial. Obstet Gynecol.

[41] Madhi S.A., Cutland C.L., Jose L., Koen A., Govender N., Wittke F. (2016). Safety and immunogenicity of an investigational maternal trivalent group B *Streptococcus* vaccine in healthy women and their infants: a randomised phase 1b/2 trial. Lancet Infect Dis.

[42] Leroux-Roels G., Maes C., Willekens J., De Boever F., de Rooij R., Martell L. (2016). A randomized, observer-blind Phase Ib study to identify formulations and vaccine schedules of a trivalent group B *Streptococcus* vaccine for use in non-pregnant and pregnant women. Vaccine.

[43] Heyderman R.S., Madhi S.A., French N., Cutland C., Ngwira B., Kayambo D. (2016). Group B *Streptococcus* vaccination in pregnant women with or without HIV in Africa: a non-randomised phase 2, open-label, multicentre trial. Lancet Infect Dis.

[44] Larsson C., Lindroth M., Nordin P., Stalhammar-Carlemalm M., Lindahl G., Krantz I. (2006). Association between low concentrations of antibodies to protein alpha and Rib and invasive neonatal group B streptococcal infection. Arch Dis Child Fetal Neonatal Ed.

[45] Boslego J.W. (2012). Profile: PATH's vaccine development global program. Hum Vaccines Immunotherapeut.

[46] Kim S.Y., Russell L.B., Park J., Verani J.R., Madhi S.A., Cutland C.L. (2014). Cost-effectiveness of a potential group B streptococcal vaccine program for pregnant women in South Africa. Vaccine.

[47] Roberts J.N., Gruber M.F. (2015). Regulatory considerations in the clinical development of vaccines indicated for use during pregnancy. Vaccine.

[48] Akanmori B. (2015). The African Vaccine Regulatory Forum (AVAREF): a platform for collaboration in a public health emergency. WHO Drug Inform.

[49] World Health Organization. Developing country vaccine regulators' network (DCVRN); 2012.

[50] U.S. Food and Drug Administration. Clinical development and requirement for licensure of vaccines intended for use during pregnancy to prevent disease in the infant; 2015.

[51] World Health Organization (2016). Global Advisory Committee on vaccine safety 15–16 June 2016. Wkly Epidemiol Rec.

[52] Madhi S.A., Dangor Z., Heath P.T., Schrag S., Izu A., Sobanjo-Ter Meulen A. (2013). Considerations for a phase-III trial to evaluate a group B *Streptococcus* polysaccharide-protein conjugate vaccine in pregnant women for the prevention of early- and late-onset invasive disease in young-infants. Vaccine.

[53] White A., Madhi S.A. (2015). Ethical considerations for designing GBS maternal vaccine efficacy trials in low-middle income countries. Vaccine.

[54] Baker C.J., Carey V., Edwards M., Ewell M., Ferrieri P., Ferris D. (2009). Women receiving group B *Streptococcus* serotype III tetanus toxoid (GBS-III-TT) vaccine have reduced vaginal and rectal acquisition of GBS type III. Infectious Diseases Society of America 47th annual meeting, Philadelphia, Pennsylvania.

[55] Goldblatt D., Ramakrishnan M., O'Brien K. (2013). Using the impact of pneumococcal vaccines on nasopharyngeal carriage to aid licensing and vaccine implementation; a PneumoCarr meeting report March 27–28, 2012, Geneva. Vaccine.

[56] O'Brien K., Binka F., Marsh K., Abramson J.S. (2016). Mind the gap: jumping from vaccine licensure to routine use. Lancet (London, England).

[57] Dellepiane N., Akanmori B.D., Gairola S., Jadhav S.S., Parker C., Rodriguez C. (2015). Regulatory pathways that facilitated timely registration of a new group A meningococcal conjugate vaccine for Africa's meningitis belt countries. Clin Infect Dis: Off Publ Infect Dis Soc Am.

[58] Dellepiane N., Wood D. (2015). Twenty-five years of the WHO vaccines prequalification programme (1987–2012): lessons learned and future perspectives. Vaccine.

